# The mechanism of Qijing Mingmu decoction on cellular senescence of conjunctivochalasis

**DOI:** 10.1186/s12906-023-04138-x

**Published:** 2023-08-29

**Authors:** Minhong Xiang, Jiang Liu, Kai Ma, Yongyi Sha, Yueping Zhan, Wei Zhang, Xueqing Kong

**Affiliations:** 1 Department of Ophthalmology, Putuo Hospital, Shanghai University of Traditional Chinese Medicine, No.164 Lanxi Road, Shanghai, 200062 P.R. China; 2 Shanghai Putuo Central School of Clinical Medicine, Anhui Medical University, Hefei, 230032 China; 3 Department of Central Lab, Putuo Hospital, Shanghai University of Traditional Chinese Medicine, Shanghai, 200062 China

**Keywords:** Conjunctivochalasis, Chinese herbal medicine, Cellular senescence, p38 MAPK signaling

## Abstract

**Background:**

Qijing Mingmu decoction (QJMM), a compound Chinese medicine preparation, which consists of Lycium barbarum, Polygonatum, Ophiopogon japonicus, Poria cocos, Glycyrrhiza, Eclipta prostrata and Ligusticum striatum, has been confirmed to be effective for the treatment of conjunctivochalasis (CCH) in clinic and reduce cellular senescence. However, the underlying mechanism is still unknown. Our previous study revealed that p38-mediated cellular senescence contributed to the pathogenesis of CCH.

**Methods:**

To explore whether p38 might be the potential therapeutic target of QJMM for CCH, CCH fibroblasts were treated with QJMM granule and then the effect of QJMM granule on the expression and promoter activity of p38α was determined by western blot and dual luciferase reporter gene assay, respectively. Meanwhile, the influence of QJMM granule on cell proliferation, oxidative stress, cellular senescence and the expression of the cellular senescence-associated genes were measured by corresponding methods.

**Results:**

QJMM granule significantly decreased the protein expression of p38α and p-p38α in CCH fibroblasts in a dose-dependent manner and inhibited p38α promoter activity. QJMM granule as well as the p38 inhibitor SB203580 reduced the level of reactive oxygen species and increased the activity of superoxide dismutase in CCH fibroblasts. QJMM granule and SB203580 promoted cell proliferation and reduced the percentage of SA-β-Gal-positive cells. The mRNA and protein expression of p53 and p21 was remarkably down-regulated by QJMM granule as well as SB203580 and that of SMP30 was up-regulated in CCH fibroblasts.

**Conclusions:**

Our findings demonstrated that QJMM granule was effective for alleviating cellular senescence of CCH fibroblasts by p38 MAPK signaling and the followed p53/p21 signaling.

## Background

As a most common ocular disorder, conjunctivochalasis (CCH) is distinguished by loosely redundant conjunctival folds located in the space of the lower eyelid and the eyeball [1]. CCH is an age-dependent ocular disease [2, 3]. Our previous community-based study revealed a prevalence rate of 44.08% for CCH in people aged no less than 60 years old [4]. Mimura et al. revealed that the prevalence of CCH dramatically increased with age in Japanese, for which the incidence increased from 6.8% to 1 to 10-years old persons to 100% for 91 to 100-years old persons [3]. Gumus K et al. demonstrated the severity of CCH were increased with age by optical coherence tomography [5]. At present, the mostly used therapies for CCH were artificial tears and surgical treatment [6, 7]. However, artificial tears can only relieve some of the symptoms and surgical treatment is traumatic with slower recovery [8–11]. Therefore, it is necessary to seek better and more effective therapies for CCH.

Chinese medical herbs have been applied in China for thousands of years for the management of diverse diseases, including different kinds of ocular disorders [12]. For example, Lycium barbarum polysaccharides protected the rats against both chronic and acute ocular hypertension [13, 14]. The clinical study showed that traditional Chinese prescription Chi-Ju-Di-Huang-Wan, which was composed of Lycii Fructus, Chrysanthemi Flos and Rehmanniae could effectively relieve the clinically relevant symptoms of dry eye [15].

Qijing Mingmu decoction (QJMM), a formula consisting of seven natural herbs, is ourselves-designed prescription for the treatment of CCH. Our clinical studies showed that QJMM could maintain the stability of tear film, improve the mucin in tear, promote the repair of damaged ocular surface tissue, and increase the tear secretion, thus having a good clinical therapeutic effect for CCH [16, 17]. Meanwhile, several herbs of QJMM had been reported to be useful for alleviating aging and inflammation. For example, as a traditional medical herb, Lycium barbarum has been used for more than two thousand years and its water extracts can increase DNA synthesis rate of the aging human embryonic lung diploid fibroblasts and prolong its life span [18]. However, the underlying mechanism of QJMM for CCH treatment is still unclear.

In view of the high incidence of CCH in the elderly, we suppose that cellular senescence might mediate the pathogenesis of CCH. Our previous study demonstrated that cellular senescence was responsible for CCH and p38 MAPK signaling and played a vital role in the cellular senescence progress in CCH fibroblasts [19, 20]. In the previous research, we also found that QJMM could significantly down-regulate MAPK signaling in CCH fibroblasts, especially p38 MAPK signaling, which was induced by TNF-α [21]. QJMM combined with artificial tears treatment also decreased cellular senescence in the conjunctival tissue of CCH patients [17]. Therefore, we hypothesize that QJMM might have an anti-cellular senescence effect via p38 MAPK signaling. To investigate whether the effect of QJMM was by affecting p38-mediated cellular senescence, CCH fibroblasts were treated with QJMM granule and then cell proliferation, cellular senescence, oxidative stress-related markers reactive oxygen species (ROS) production and superoxide dismutase (SOD) activity, and the expression of senescence-associated genes were determined using the corresponding methods.

## Methods

### Ethics statement

The protocol was approved by Ethics Committee of Putuo Hospital, Shanghai University of Traditional Chinese Medicine (Ethic code: PTEC-A-2021-14-1). The written informed consents were supplied by each patient.

### Fibroblasts culture

The CCH fibroblasts and normal fibroblasts were obtained as the previous method [19]. The cells were cultured in Dulbecco’s Modified Eagle’s Medium (DMEM) supplied with 10% fetal bovine serum and 1% penicillin-streptomycin at 37 °C and 5% CO_2_.

### Preparation of QJMM granule

QJMM was prepared with *Lycium barbarum* L. (15 g), *Polygonatum acuminatifolium* Kom. (20 g), *Ophiopogon japonicus* (Thunb.) Ker Gawl. (20 g), *Poria cocos* (Schw.) Wolf (10 g), *Glycyrrhiza uralensis* Fisch. (3 g), *Eclipta prostrata* (L.) L. (15 g) and *Ligusticum striatum* DC. (3 g) (Table 1). QJMM granule was processed from the previous study [21].

**Table 1:** The compositions of QJMM

Latin name	Chinese name	Daily adult dose (g)
*Lycium barbarum* L.	Gouqi	15
*Polygonatum acuminatifolium* Kom.	Huangjing	20
*Ophiopogon japonicus* (Thunb.) Ker Gawl.	Maidong	20
*Poria cocos* (Schw.) Wolf	Fuling	10
*Glycyrrhiza uralensis* Fisch.	Zhigancao	3
*Eclipta prostrata* (L.) L.	Hanliancai	15
*Ligusticum striatum* DC.	Chuanxiong	3

### Experimental design

To investigate the effect of QJMM granule on p38 MAPK signaling in CCH fibroblasts, the fibroblasts derived from the loose conjunctival tissue of CCH patients were treated with different concentration of QJMM granule (0, 0.805, 1.61, 3.22 mg/L). In the other experiments, the fibroblasts were divided into three groups: CCH, CCH + QJMM (CCH fibroblasts treated with 1.61 mg/L Qijing Mingmu decoction granule) and CCH + SB203580 (CCH fibroblasts treated with p38 inhibitor SB203580). The normal fibroblasts were applied as controls.

### CCK-8 assay

The viability of fibroblasts was measured using a Cell Counting Kit-8 (CP002, SAB, Sioux Falls, SD, USA) according to the manufacturer’s instructions. A volume of 100 µl fibroblast suspension (about 3 × 10^3^ cells) was added into a 96-well plate. The work solution was prepared by mixing CCK-8 reagent with serum-free DMEM medium by a volume ration of 1:10. After 12 h, 24 or 48 h of cultivation, 100 µl of work solution was added into each well. The fibroblasts were then incubated for another hour at 37 °C and 5% CO_2_. The absorbance of mixture in each well under 450 nm wavelength was measured and recorded.

### ROS level and SOD activity detection

The intracellular ROS production was determined using a ROS Assay Kit (S0033, Beyotime) and SOD activity using a SOD Test Kit (A0001, Nanjing Jiancheng Bioengineering Institute, Jiangsu, China) according to the responding manufacture’s instruction, respectively.

### SA-β-Gal staining

SA-β-Gal staining for the fibroblasts was carried out with SA-β-Gal Staining Kit using the previous method [19]. Briefly, the collected fibroblasts were fixed using the β-Gal staining fixative at room temperature for 15 min. The fibroblasts were rinsed with phosphate buffer saline for 3 times and then incubated at 37 °C overnight with the prepared β-Gal staining work solution, which was prepared according to the manufacture’s instruction. The stained fibroblasts were observed under an optical microscope.

### Western blot

The total protein of fibroblasts was extracted and quantified with a bicinchoninic acid protein assay kit (Nanjing Jiancheng Bioengineering Institute, Nanjing, Jiangsu, China). After separation with electrophoresis on a 10% or 12% polyacrylamide gel, the protein was transferred onto a polyvinylidene fluoride membrane. The interested p38α-membrane was blocked using 5% bovine serum albumin and the other membranes using 5% non-fat dry milk at room temperature for 1 h. Then, the membranes were incubated with the followed primary antibodies: p38α (#9212, 1:1000), p-p38 (#9211, 1:1000), p53 (#9282, 1:1000), p21 (#2947, 1:1000), SMP30 (Ab233007, 1:400) and GAPDH (#5174, 1:2000) at 4℃ overnight. Subsequently, the membranes were incubated with a secondary antibody (goat anti-rabbit IgG, A0208, 1:1000) for 1 h at 37℃. After coloration with electrochemiluminescence solution, the protein bands were scanned using a Tanon-5200 instrument (Shanghai Tianneng Technology Co., Ltd, Shanghai, China).

### Quantitative real-time PCR (qRT-PCR)

The fibroblast RNA was extracted using a Trizol method. The cDNA was synthesized with a Revert Aid First Strand cDNA Synthesis Kit (K1622, Thermo Scientific, Waltham, MA, USA). The expression of p38α, p53, p21 and SMP30 was measured using a SYBR Green qPCR kit (K0223, Thermo Scientific) on an ABI-7300 system. GAPDH was selected as a reference gene. The previous primer sequence was used here [19]. The relative mRNA expression was calculated using a 2 ^–ΔΔct^ method.

### Dual luciferase reporter gene assay

The p38 promoter sequence was inserted into pGL3-Enhancer vector, which contained Luc2 and hRluc genes, using the followed primer sequences:

- p p38-F: 5’ -CCCTCGAGCCTGCCTTTGGGGAGG-3’ (Xho I),
- p p38-R: 5’-CCAAGCTTCAGACGAAGGGCGCCAG-3’ (Hind III).

The obtained pGL3-Enhancer-p-p38 was transfected into the fibroblasts. Six hours after transfection, the fibroblasts were treated with 1.61 mg/L QJMM granule or not. Twenty–four hours after transfection, the fibroblasts were treated using the Dual-Luciferase® Reporter(DLR™)Assay System (E1910, Promega, Madison, WI, USA) according to the manufacturer’s instruction. The firefly and luciferase renilla activity intensity were detected, respectively.

### Statistical analysis

The experimental data was presented as mean ± standard deviation. The difference between two groups was assessed using student-t test and the difference among multiple comparisons among three or more groups using one-way ANOVA followed by Turkey’s test. *P* < 0.05 was considered as with significant difference.

## Results

### Effects of QJMM on p38 MAPK signaling in CCH fibroblasts

**Fig. 1:**
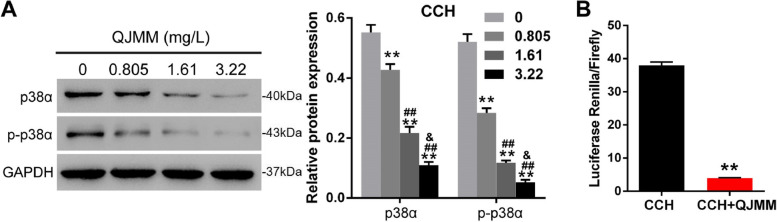
QJMM granule affected p38 MAPK signaling in CCH fibroblasts. **A** The protein expression of p38α and p-p38α in CCH fibroblasts treated with different concentration of QJMM granule. **B** The promoter activity of p38α in CCH fibroblasts treated with 1.61 mg/L QJMM. **P < 0.001 compared with CCH group; ^##^ *P* < 0.001 compared with CCH + 0.805 mg/L QJMM group; ^&^ *P* < 0.05 compared with CCH + 1.61 mg/L QJMM group; *n* = 3

Our previous research has demonstrated that p38 MAPK signaling participants in the cellular senescence progress of CCH fibroblast and thus implicates in the development of CCH [19]. To investigate whether QJMM affect CCH by p38 MAPK signaling, the effect of QJMM granule on the expression of p38α and p-p38α in CCH fibroblasts was firstly measured by western blot. Both the total p38α and p-p38α protein expression was significantly decreased by QJMM granule in a dose-dependent manner (Fig. 1A). Thus, the concentration of 1.61 mg/L QJMM granule was used in the following experiments. The dual luciferase reporter gene assay confirmed that QJMM granule remarkably reduced the activity of p38 promoter in CCH fibroblasts (Fig. 1B). These results indicate the effective manipulation of QJMM on p38 MAPK signaling.

### Effects of QJMM on the levels of ROS and SOD activity in CCH fibroblasts

**Fig. 2:**
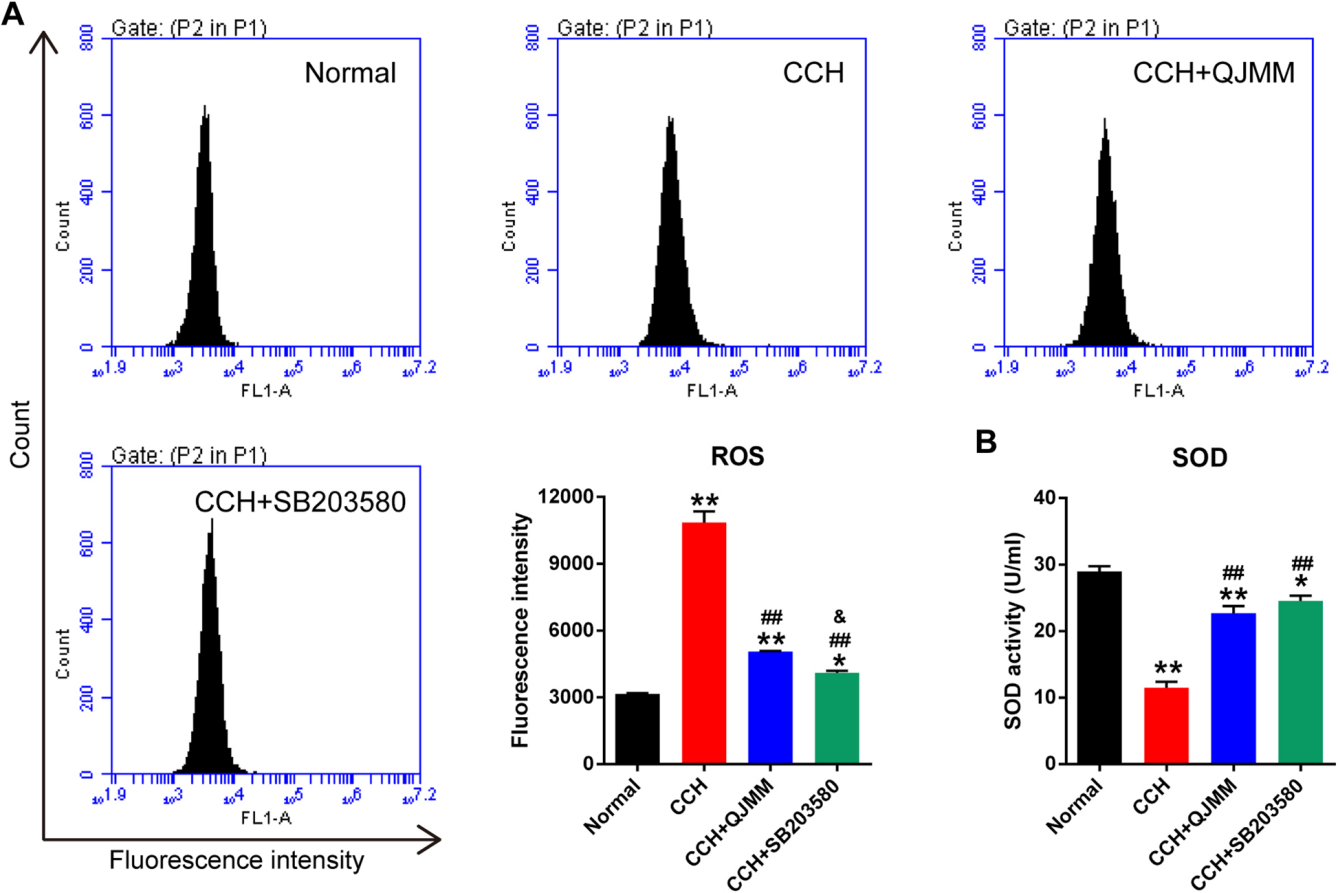
QJMM granule affected oxidative stress in CCH fibroblasts. **A** Reactive oxygen species (ROS) level in CCH fibroblasts treated with QJMM granule. **B** Superoxide dismutase (SOD) activity in CCH fibroblasts treated with QJMM granule. *P < 0.05, **P < 0.001 compared with normal group; ^##^ *P* < 0.001 compared with CCH group; ^&^ *P* < 0.05 compared with CCH + QJMM group; *n* = 3

Oxidative stress is closely related to CCH [22]. Compared with the normal fibroblasts, CCH fibroblasts had significantly higher ROS production and lower SOD activity (Fig. 2). QJMM granule, as well as p38 inhibitor SB203580, obviously decreased the ROS production and increased SOD activity in CCH fibroblasts. All these findings suggest that QJMM attenuates oxidative stress in CCH fibroblasts.

### Effects of QJMM on cell proliferation of CCH fibroblasts

**Fig. 3:**
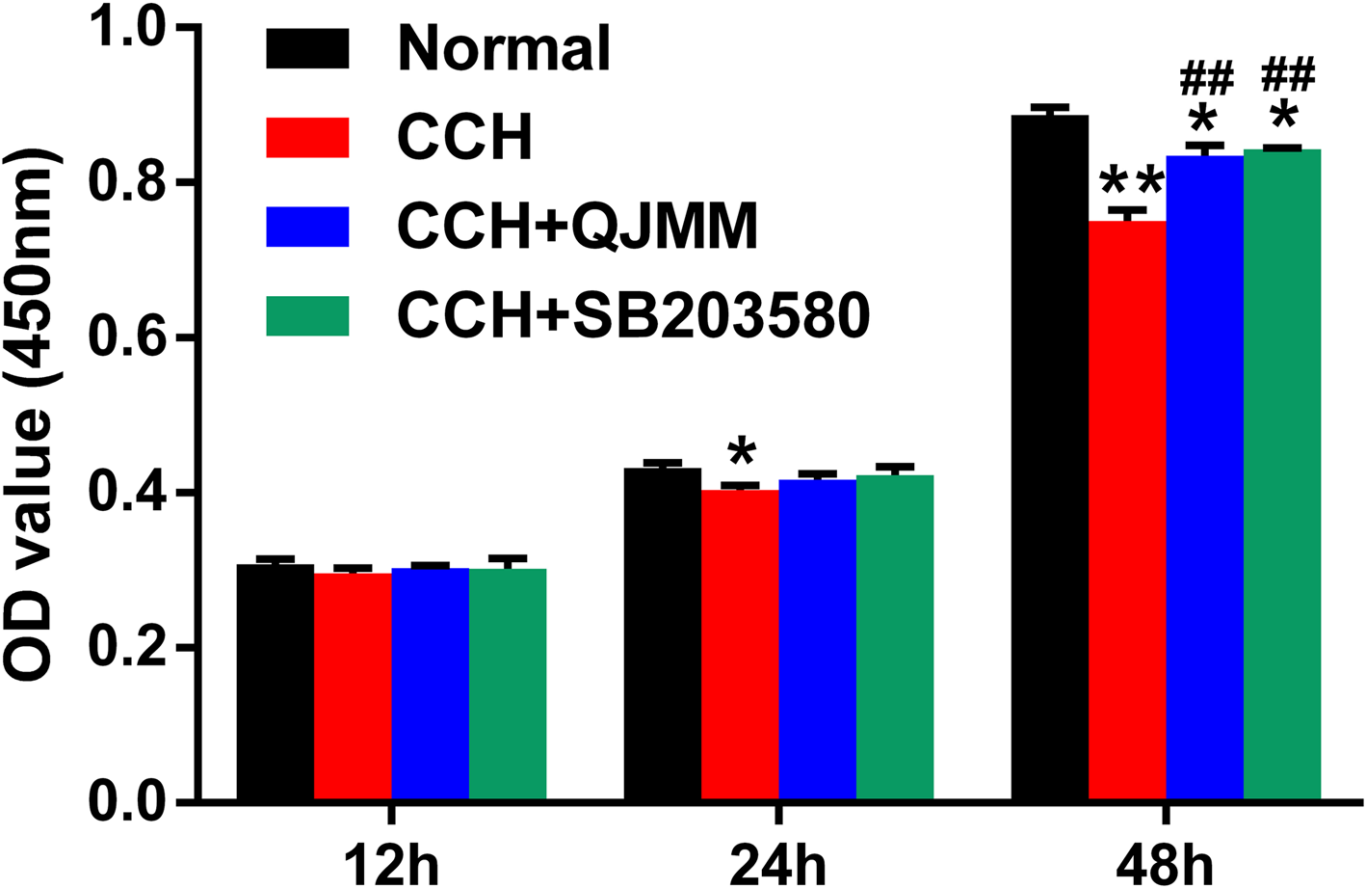
QJMM granule affected cell proliferation of CCH fibroblasts. *P < 0.05, **P < 0.001 compared with normal group; ^##^ *P* < 0.001 compared with CCH group; *n* = 3

The effect of QJMM granule on cell proliferation of CCH fibroblasts was determined by CCK-8 assay. After 24 and 48 h of cultivation, CCH fibroblasts represented significantly decreased cell viability when compared with the normal fibroblasts (Fig. 3). QJMM granule and p38 inhibitor SB203580 remarkably increased the cell viability of CCH fibroblasts.

### Effects of QJMM on cellular senescence of CCH fibroblasts

**Fig. 4:**
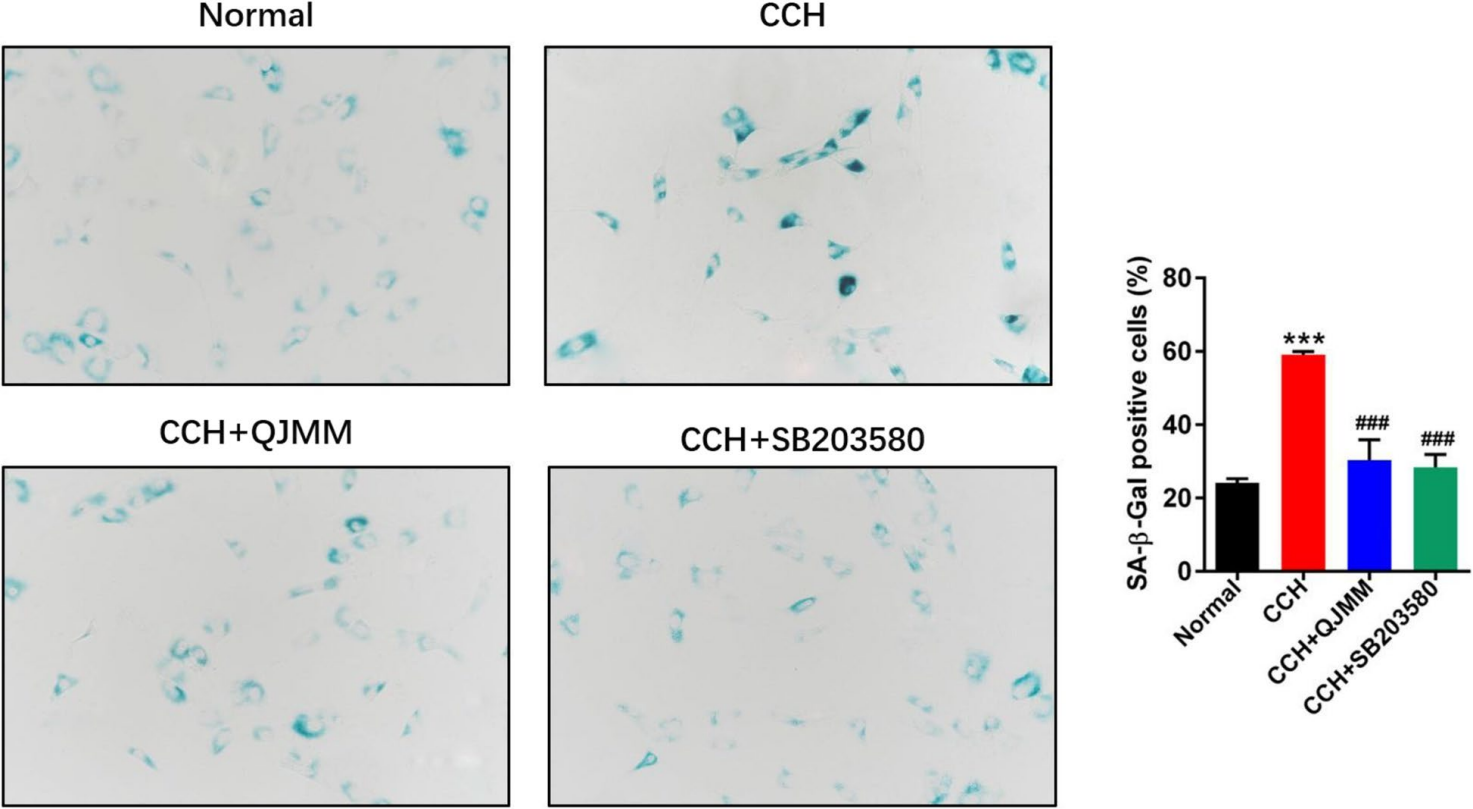
QJMM granule affected cellular senescence in CCH fibroblasts. **P < 0.001 compared with normal group; ^##^ *P* < 0.001 compared with CCH group; *n* = 3

Consistent with the previous study [19], CCH fibroblasts had obviously more senescent cells than the normal fibroblasts did, which was evidenced by SA-β-Gal staining (Fig. 4). QJMM granule significantly reduced the percentage of SA-β-Gal positive cells, which was similar with the effect of p38 inhibitor SB203580.

### Molecular mechanism of QJMM in CCH fibroblasts

**Fig. 5:**
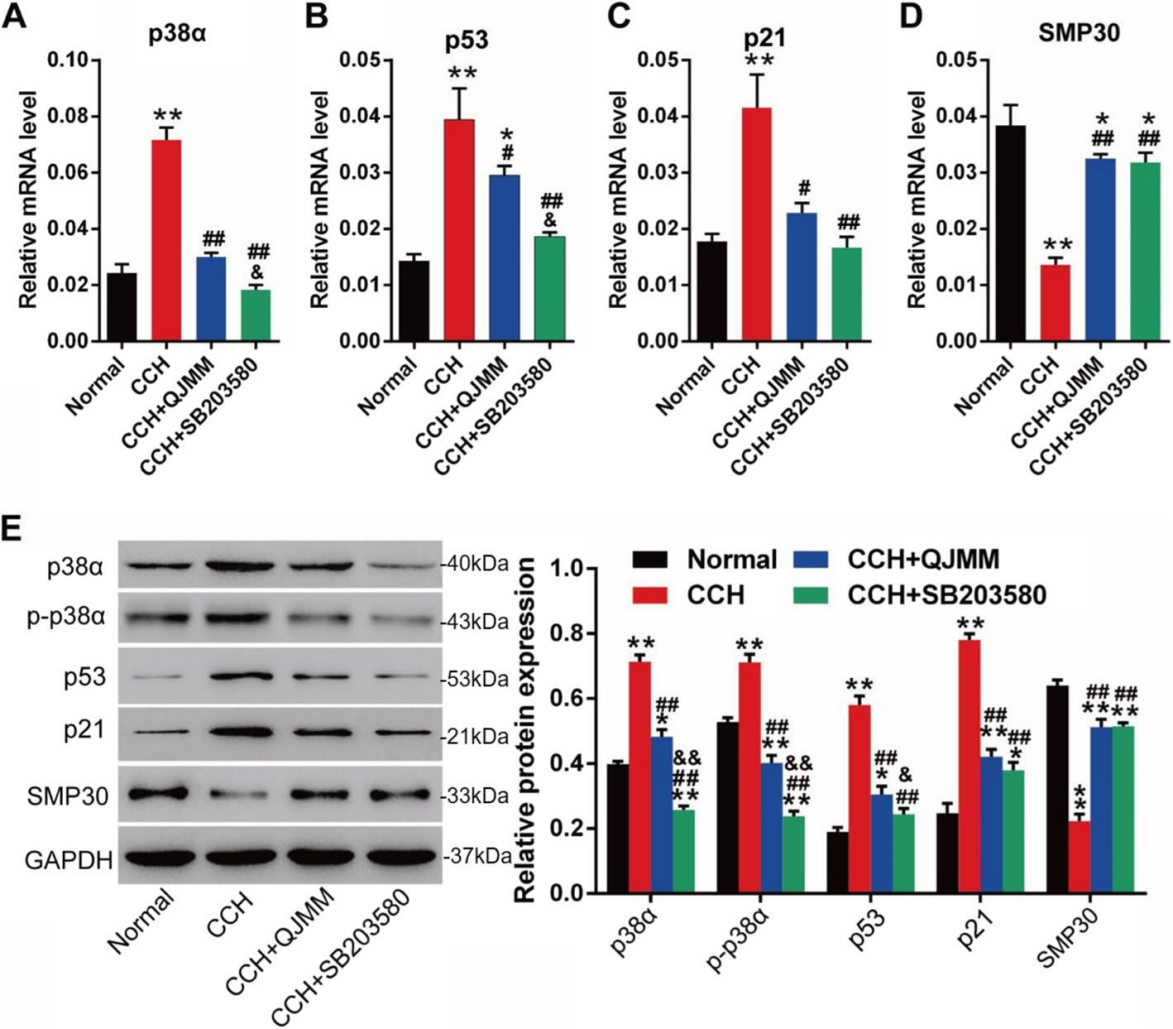
The mRNA (**A**-**D**) and protein (**E**) levels of p38 and senescence-associated genes in CCH fibroblasts treated with QJMM granule. *P < 0.05, **P < 0.001 compared with normal group; ^#^ *P* < 0.05, ^##^ *P* < 0.001 compared with CCH group; ^&^ *P* < 0.05, ^&&^ *P* < 0.001 compared with CCH + QJMM group; *n* = 3

**Fig. 6:**
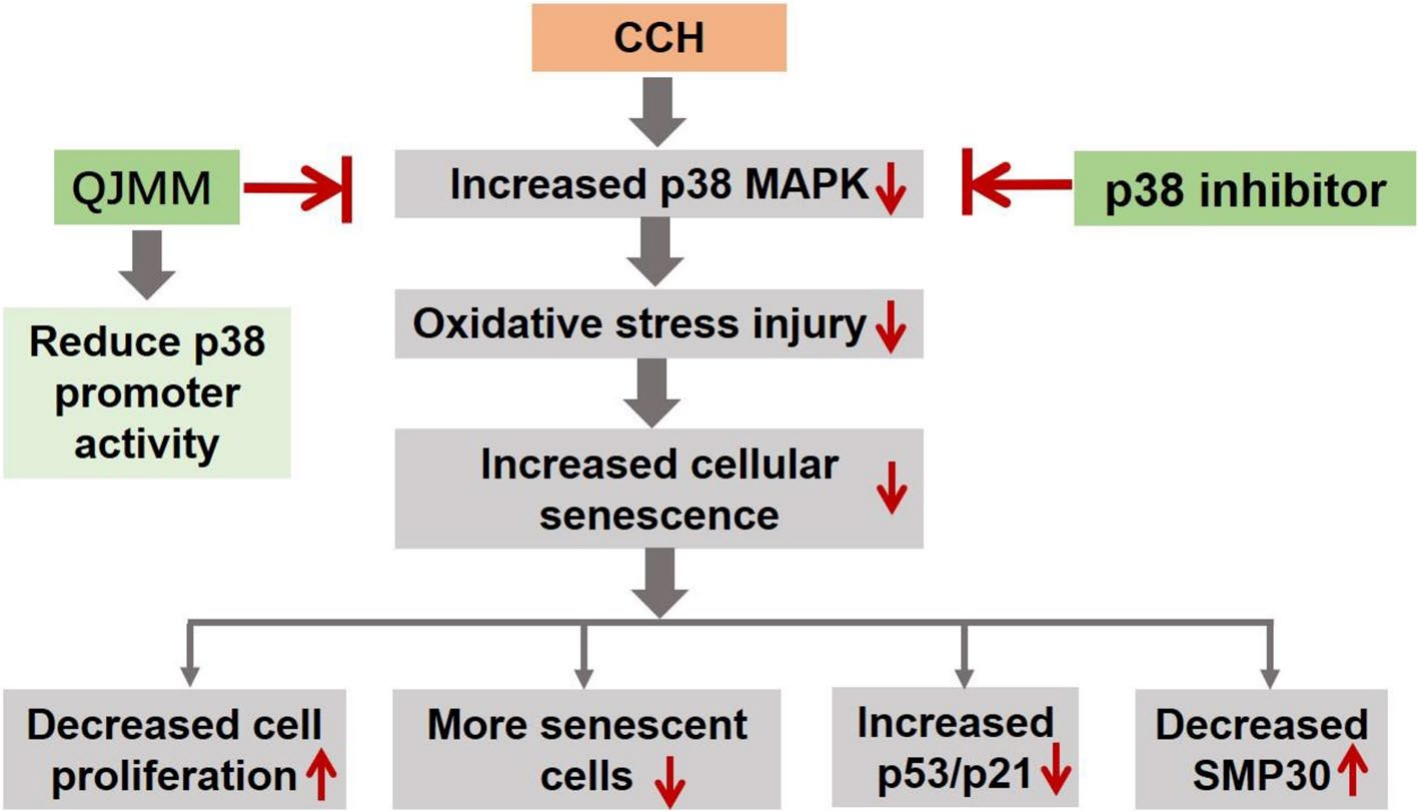
Molecular mechanism of QJMM on cellular senescence of CCH

To explore whether the effect of QJMM granule on cellular senescence of CCH fibroblast was through p38 MAPK signaling, the expression of senescence-associated genes (p53, p21 and SMP30) and p38 signaling was measured. The results showed that p38α mRNA, total p38α protein and p-p38α protein in CCH fibroblasts were significantly enhanced compared with those in the normal control (Fig. 5A and E). QJMM granule and p38 inhibitor SB203580 remarkably blocked the p38 signaling. The increased expression of pro-senescent genes p53 and p21 was significantly down-regulated by QJMM granule and p38 inhibitor SB203580, while the decreased expression of anti-senescent gene SMP30 in CCH fibroblasts was significantly up-regulated (Fig. 5B-E). Molecular mechanism of QJMM on cellular senescence of CCH was shown in Fig. 6.

## Discussion

Our previous study demonstrated that Qijing Mingmu decoction (QJMM) drug serum could significantly suppress the expression of MMP1, MMP3 and TIMP1, and increase the expression of TIMP3 and TGS-6 in CCH fibroblasts, indicating the effectiveness of QJMM drug serum for the therapy of CCH [23, 24]. Due to the drug serum that was prepared by metabolism in vivo after intragastomy in SD rats, the composition of serum was complex and the concentration of the drug in serum was uncertain. It is difficult to identify the effective factors. Therefore, Qijing Mingmu decoction granule was applied to directly treat CCH fibroblasts here. The results showed that QJMM granule significantly down-regulated the expression and promoter activity of p38α. Our previous study has revealed that p38-mediated cellular senescence contribute to the pathogenesis of CCH [19]. Therefore, we supposed that the therapeutical effect of QJMM might be at least partly through p38-mediated cellular senescence.

It has been confirmed that oxidative stress damage, inflammation and aging were present in CCH [5, 11, 22, 25, 26]. Diverse bioactive components in QJMM had biological effects, such as anti-aging, anti-oxidation and anti-inflammation. The extracts of Lycium barbarum L had the healthy benefits of antioxidant, anti-aging, anti-fatigue and improving eyesight [27–30]. The aqueous extract of Polygonatum showed anti-inflammatory effects in a mouse model of ear edema and protected endothelial progenitor cells in nature senescent rats via enhancing antioxidant capacity and telomerase activity without obvious genetic toxicity [31–33]. Ophiopogon japonica had been used for curing inflammation-related diseases and its bioactive component protected against diabetic nephropathy by inhibiting oxidative stress and inflammation [34, 35]. Poria cocos polysaccharides, the major active ingredient of Poria cocos, had biological effects of anti-aging, anti-oxidation and anti-inflammation and it represented hepatoprotective effects by inhibiting cell apoptosis and inflammatory response [36, 37].

The further experiments showed that QJMM granule had the similar effects with p38 inhibitor SB203580 on oxidative stress, cell proliferation and cellular senescence in CCH fibroblasts. QJMM affected oxidative stress by reducing ROS level and increasing SOD activity in CCH fibroblasts. Meanwhile, QJMM granule also promoted the proliferation of CCH fibroblasts and inhibited cellular senescence. All these findings revealed the important role of QJMM granule in the treatment of CCH. P38 MAPK was highly expressed in CCH tissue and fibroblasts compared with that in normal controls. Cellular senescence was mediated by p38/p53/p21 signaling axis in CCH progress. Lycium bararum polysaccharides can effectively inhibit senescence of human umbilical vein endothelial cells by decreasing the expression of p53 and p16 [38]. Here, QJMM granule decreased the expression of p53 and p21, and increased SMP30 expression as well as p38 inhibitor SB203580 in the CCH fibroblasts. All these indicate the effects of QJMM in cellular senescence in CCH fibroblasts might or partly be dependent on p38/p53/p21 signaling axis.

## Conclusions

In conclusion, we investigated the effects of Qijing Mingmu decoction granule on CCH cellular senescence. The results showed that Qijing Mingmu decoction granule could effectively alleviate CCH cellular senescence via blocking p38 MAPK signaling pathway. This study will be benefit for better understanding of the mechanism of CCH and may provide a reference for researching traditional Chinese medicine theory.

## Data Availability

The datasets used and/or analyzed during the current study are available from the corresponding author on reasonable request.

## Abbreviations

QJMM: Qijing Mingmu decoction
CCH: Conjunctivochalasis
ROS: Reactive oxygen species
SOD: Superoxide dismutase
SMP30: Senescence marker protein-30
DMEM: Dulbecco’s Modified Eagle’s Medium
qRT-PCR: Quantitative real-time PCR

## References

[1] Meller D, Tseng SC (1998). Conjunctivochalasis: literature review and possible pathophysiology. Surv Ophthalmol.

[2] Di Pascuale MA, Espana EM, Kawakita T, Tseng SC (2004). Clinical characteristics of conjunctivochalasis with or without aqueous tear deficiency. Br J Ophthalmol.

[3] Mimura T, Yamagami S, Usui T, Funatsu H, Mimura Y, Noma H (2009). Changes of conjunctivochalasis with age in a hospital-based study. Am J Ophthalmol.

[4] Zhang X, Li Q, Zou H, Peng J, Shi C, Zhou H (2011). Assessing the severity of conjunctivochalasis in a senile population: a community-based epidemiology study in Shanghai, China. BMC Public Health.

[5] Gumus K, Pflugfelder SC (2013). Increasing prevalence and severity of conjunctivochalasis with aging detected by anterior segment optical coherence tomography. Am J Ophthalmol.

[6] Otaka I, Kyu N (2000). A new surgical technique for management of conjunctivochalasis. Am J Ophthalmol.

[7] Kiss HJ, Németh J (2015). Isotonic glycerol and sodium hyaluronate containing artificial tear decreases conjunctivochalasis after one and three months: a self-controlled, unmasked study. PLoS ONE.

[8] Zhang XR, Zhang ZY, Hoffman MR (2012). Electrocoagulative surgical procedure for treatment of conjunctivochalasis. Int Surg.

[9] Petris CK, Holds JB (2013). Medial conjunctival resection for tearing associated with conjunctivochalasis. Ophthalmic Plast Reconstr Surg.

[10] Wang H, Gao F, Pan YZ (2016). The treatment outcomes of crescent-shaped conjunctiva resection combined with conjunctiva sclera fixation for severe conjunctivochalasis. Eur Rev Med Pharmacol Sci.

[11] Marmalidou A, Kheirkhah A, Dana R (2018). Conjunctivochalasis: a systematic review. Surv Ophthalmol.

[12] Sepahi S, Ghorani-Azam A, Hossieni SM, Mohajeri SA, Khodavrdi E (2021). Pharmacological effects of saffron and its constituents in ocular disorders from in vitro studies to clinical trials; a systematic review. Curr Neuropharmacol.

[13] Lakshmanan Y, Wong FS, Yu WY, Li SZ, Choi KY, So KF (2019). Lycium Barbarum polysaccharides rescue neurodegeneration in an acute ocular hypertension rat model under pre- and posttreatment conditions. Invest Ophthalmol Vis Sci.

[14] Lakshmanan Y, Wong FSY, Zuo B, So KF, Bui BV, Chan HH (2019). Posttreatment intervention with Lycium Barbarum polysaccharides is neuroprotective in a rat model of chronic ocular hypertension. Invest Ophthalmol Vis Sci.

[15] Chao WW, Tan SQ, Liu JH, Chen MM, Shiu HW, Chao HM (2020). Dry Eye: the effect of Chi-Ju-Di-Huang-Wan plus Si Wu Tang and the underlying mechanism. J Altern Complement Med.

[16] Xiang MH, Rao YM, Li QS, Zhang XR, Zhou HM, Han ZM (2012). Tear functional changes of JingQiMingMu decoction for conjunctivochalasis. Rec Adv Ophthalmol.

[17] Zhou HM, Xiang MH, Ma K, Wen H, Zhao YQ, Liu J (2020). Effects of Qi Jing Mingmu decoction combined with artificial tears on the clinical results and cell aging of conjunctivochalasis. Guoji Yanke Zazhi (Int Eye Sci).

[18] Wu BY, Zou JH, Meng SC (2003). Effect of wolfberry fruit and epimedium on DNA synthesis of the aging-youth 2BS fusion cells. Zhongguo Zhong Xi Yi Jie He Za Zhi.

[19] Xiang M, Mo L, Zhan Y, Wen H, Zhou H, Miao W (2019). P38-mediated cellular senescence in conjunctivochalasis fibroblasts. Invest Ophthalmol Vis Sci.

[20] Xiang M, Zhang W, Wen H, Mo L, Zhao Y, Zhan Y (2019). Comparative transcriptome analysis of human conjunctiva between normal and conjunctivochalasis persons by RNA sequencing. Exp Eye Res.

[21] Jia YL, Xiang MH, Wen H, Li QS, Zhan YP, Huang L (2018). Effect of Qijingmingmu decoction granule on MAPK signal pathway in fibroblasts of conjunctivochalasis stimulated by TNF-α. Rec Adv Ophthalmol.

[22] Ward SK, Wakamatsu TH, Dogru M, Ibrahim OM, Kaido M, Ogawa Y (2010). The role of oxidative stress and inflammation in conjunctivochalasis. Invest Ophthalmol Vis Sci.

[23] Xiang MH, Li YJ, Zhang XR, Li QS, Han ZM, Zhang L (2013). Effects of qijingmingmu soup on the expression of matrix metalloproteinases in the conjuntival fibroblasts of conjunctivochalasis. Chin J Exp Ophthalmol.

[24] Ke MQ, Zhang XR, Xiang MH, Li QS, Wang HM, Zhou GZ (2017). Effects of Qijing Mingmu decoction on expression of TSG-6 and PTX3 in conjunctivochalasis. Jounal of Traditional Chinese Ophthalmology.

[25] Erdogan-Poyraz C, Mocan MC, Bozkurt B, Gariboglu S, Irkec M, Orhan M (2009). Elevated tear interleukin-6 and interleukin-8 levels in patients with conjunctivochalasis. Cornea.

[26] Acera A, Vecino E, Duran JA (2013). Tear MMP-9 levels as a marker of ocular surface inflammation in conjunctivochalasis. Invest Ophthalmol Vis Sci.

[27] Zhang Q, Chen W, Zhao J, Xi W (2016). Functional constituents and antioxidant activities of eight chinese native goji genotypes. Food Chem.

[28] Yao R, Heinrich M, Weckerle CS (2018). The genus Lycium as food and medicine: a botanical, ethnobotanical and historical review. J Ethnopharmacol.

[29] Gao Y, Wei Y, Wang Y, Gao F, Chen Z (2017). Lycium Barbarum: a traditional chinese herb and a promising anti-aging agent. Aging Dis.

[30] Tian X, Liang T, Liu Y, Ding G, Zhang F, Ma Z (2019). Extraction, structural characterization, and biological functions of Lycium Barbarum polysaccharides: a review. Biomolecules.

[31] Xian YF, Lin ZX, Xu XY, Su ZR, Chen JN, Lai XP (2012). Effect of Rhizoma Polygonati on 12-O-tetradecanoylphorbol-acetate-induced ear edema in mice. J Ethnopharmacol.

[32] Zai QQ, Qin Z, Ye L (2016). Effect of Rhizoma Polygonati on the function of endothelial progenitor cells and telomerase activity in nature senescent rats. Zhongguo Zhong Xi Yi Jie He Za Zhi.

[33] Chen H, Feng R, Guo Y, Sun L, Zhou Y, Jiang J (2001). Toxicity studies of Rhizoma Polygonati Odorati. J Ethnopharmacol.

[34] Zhao JW, Chen DS, Deng CS, Wang Q, Zhu W, Lin L (2017). Evaluation of anti-inflammatory activity of compounds isolated from the rhizome of Ophiopogon japonicas. BMC Complement Altern Med.

[35] Qiao Y, Jiao H, Wang F, Niu H (2020). Ophiopogonin D of Ophiopogon japonicus ameliorates renal function by suppressing oxidative stress and inflammatory response in streptozotocin-induced diabetic nephropathy rats. Braz J Med Biol Res.

[36] Wu K, Fan J, Huang X, Wu X, Guo C (2018). Hepatoprotective effects exerted by Poria cocos polysaccharides against acetaminophen-induced liver injury in mice. Int J Biol Macromol.

[37] Li X, Ma L, Zhang L (2019). Molecular basis for Poria cocos mushroom polysaccharide used as an antitumor drug in China. Prog Mol Biol Transl Sci.

[38] Liu L, Wang XN, Liu Z, Wang LN, Wu J, Wang W (2011). Effect of lycium bararum polysaccharides on angiotensin II-induced senescence of human umbilical vein endothelial cells and expressions of P53 and P16. Nan Fang Yi Ke Da Xue Xue Bao.

